# Supplementary material for: Clinical characteristics of treatment-resistant depression in adults in Hungary: Real-world evidence from a 7-year-long retrospective data analysis
Source: PLoS One. 2021 Jan 20;16(1):e0245510. doi: 10.1371/journal.pone.0245510 (PMC7817060; doi:10.1371/journal.pone.0245510)
Supplement: S2 File — (DOCX) [file pone.0245510.s002.docx]

**S2 Table. Excluded diagnoses list by ICD-10 code.**

| **ICD-10 code** | **Description** |
| --- | --- |
| A81 | Atypical virus infections of central nervous system |
| E75 | Disorders of sphingolipid metabolism and other lipid storage disorders |
| F01 | Vascular dementia |
| F02 | Dementia in other diseases classified elsewhere |
| F03 | Unspecified dementia |
| F04 | Organic amnesic syndrome, not induced by alcohol and other psychoactive substances |
| F05 | Delirium, not induced by alcohol and other psychoactive substances |
| F06 | Other mental disorders due to brain damage and dysfunction and to physical disease |
| F07 | Personality and behavioural disorders due to brain disease, damage and dysfunction |
| F10 | Mental and behavioural disorders due to use of alcohol |
| F19 | Mental and behavioural disorders due to multiple drug use and use of other psychoactive substances |
| F20 | Schizophrenia |
| F22 | Persistent delusional disorders |
| F23 | Acute and transient psychotic disorders |
| F24 | Induced delusional disorder |
| F25 | Schizoaffective disorders |
| F28 | Other nonorganic psychotic disorders |
| F29 | Unspecified nonorganic psychosis |
| F30 | Manic episode |
| F31 | Bipolar affective disorder |
| G10 | Huntington’s disease |
| G30 | Alzheimer’s disease |
| G31 | Other degenerative diseases of nervous system, not elsewhere classified |
| G93 | Other disorders of brain |
| G94 | Other disorders of brain in diseases classified elsewhere |
| R41 | Other symptoms and signs involving cognitive functions and awareness |

ICD, International Classification of Diseases and Related Health Problems.

**S3 Table. Excluded medications list.**

| **ATC code** | **Active substance** |
| --- | --- |
| N03AA02 | phenobarbital |
| N03AA03 | primidone |
| N03AB02 | phenytoin |
| N03AD01 | ethosuximide |
| N03AE01 | clonazepam |
| N03AF01 | carbamazepine |
| N03AF02 | oxcarbazepine |
| N03AF03 | rufinamide |
| N03AG01 | valproic acid |
| N03AG04 | vigabatrin |
| N03AX03 | sultiame |
| N03AX09 | lamotrigine |
| N03AX10 | felbamate |
| N03AX11 | topiramate |
| N03AX12 | gabapentin |
| N03AX14 | levetiracetam |
| N03AX15 | zonisamide |
| N03AX16 | pregabalin |
| N03AX18 | lacosamide |
| N03AX21 | retigabine |
| N05AE04 | ziprasidone |
| N05AH02 | clozapine |
| N05AH03 | olanzapine |
| N05AH04 | quetiapine |
| N05AL01 | sulpiride |
| N05AL03 | tiapride |
| N05AL05 | amisulpride |
| N05AX08 | risperidone |
| N05AX12 | aripiprazole |
| N05AX13 | paliperidone |
| N05BE01 | buspirone |
| N05AN01 | lithium |
| H03AA01 | levothyroxine sodium |
| H03AA03 | Combinations of levothyroxine and liothyronine |

Medications are only used as exclusion criteria in the baseline period (180 days preceding the MDD index date).

ATC, Anatomical Therapeutic Chemical.

**S4 Table. Antidepressants categorised by drug class.**

| **ATC code** | **Active substance** | **Class** |
| --- | --- | --- |
| N06AA02 | imipramine | TCA |
| N06AA04 | clomipramine | TCA |
| N06AA06 | trimipramine | TCA |
| N06AA09 | amitriptyline | TCA |
| N06AA21 | maprotiline | TCA |
| N06AB03 | fluoxetine | SSRI |
| N06AB04 | citalopram | SSRI |
| N06AB05 | paroxetine | SSRI |
| N06AB06 | sertraline | SSRI |
| N06AB08 | fluvoxamine | SSRI |
| N06AB10 | escitalopram | SSRI |
| N06AG02 | moclobemide | MAOI |
| N06AX16 | venlafaxine | SNRI |
| N06AX21 | duloxetin | SNRI |
| N06AX03 | mianserin | OTHER |
| N06AX05 | trazodone | OTHER |
| N06AX11 | mirtazapine | OTHER |
| N06AX12 | bupropion | OTHER |
| N06AX14 | tianeptine | OTHER |
| N06AX18 | reboxetine | OTHER |
| N06AX22 | agomelatine | OTHER |
| N06AX26 | vortioxetine | OTHER |

ATC, Anatomical Therapeutic Chemical.

**S5 Table. Add-on medications categorised by drug class.**

| **ATC code** | **Active substance** | **Class** |
| --- | --- | --- |
| N03AA02 | phenobarbital | ANTIE |
| N03AA03 | primidone | ANTIE |
| N03AB02 | phenytoin | ANTIE |
| N03AD01 | ethosuximide | ANTIE |
| N03AE01 | clonazepam | ANTIE |
| N03AF01 | carbamazepine | ANTIE |
| N03AF02 | oxcarbazepine | ANTIE |
| N03AF03 | rufinamide | ANTIE |
| N03AG01 | valproic acid | ANTIE |
| N03AG04 | vigabatrin | ANTIE |
| N03AX03 | sultiame | ANTIE |
| N03AX09 | lamotrigine | ANTIE |
| N03AX10 | felbamate | ANTIE |
| N03AX11 | topiramate | ANTIE |
| N03AX12 | gabapentin | ANTIE |
| N03AX14 | levetiracetam | ANTIE |
| N03AX15 | zonisamide | ANTIE |
| N03AX16 | pregabalin | ANTIE |
| N03AX18 | lacosamide | ANTIE |
| N03AX21 | retigabine | ANTIE |
| N05AE04 | ziprasidone | AANTIP |
| N05AH02 | clozapine | AANTIP |
| N05AH03 | olanzapine | AANTIP |
| N05AH04 | quetiapine | AANTIP |
| N05AL01 | sulpiride | AANTIP |
| N05AL03 | tiapride | AANTIP |
| N05AL05 | amisulpride | AANTIP |
| N05AX08 | risperidone | AANTIP |
| N05AX12 | aripiprazole | AANTIP |
| N05AX13 | paliperidone | AANTIP |
| N05BE01 | buspirone | BUSP |
| N05AN01 | lithium | Lithium |
| H03AA01 | levothyroxine sodium | T3/T4 |
| H03AA03 | combinations of levothyroxine and liothyronine | T3/T4 |

ATC, Anatomical Therapeutic Chemical.

**S6 Table. Comorbidities analysed by ICD-10 code.**

| **ICD-10 code** | **Description** | **Comorbidity group** |
| --- | --- | --- |
| F40 | Phobic anxiety disorders | Neurotic, stress-related and somatoform disorders |
| F41 | Other anxiety disorders | Neurotic, stress-related and somatoform disorders |
| F42 | Obsessive-compulsive disorder | Neurotic, stress-related and somatoform disorders |
| F43 | Reaction to severe stress, and adjustment disorders | Neurotic, stress-related and somatoform disorders |
| F44 | Dissociative (conversion) disorders | Neurotic, stress-related and somatoform disorders |
| F45 | Somatoform disorders | Neurotic, stress-related and somatoform disorders |
| F48 | Other neurotic disorders | Neurotic, stress-related and somatoform disorders |
| E10 | Insulin-dependent diabetes mellitus | Autoimmune disease |
| G35 | Multiple sclerosis | Autoimmune disease |
| H20 | Iridocyclitis | Autoimmune disease |
| K50 | Crohn's disease | Autoimmune disease |
| K51 | Ulcerative colitis | Autoimmune disease |
| K73 | Chronic hepatitis, not elsewhere classified | Autoimmune disease |
| L10 | Pemphigus | Autoimmune disease |
| L12 | Pemphigoid | Autoimmune disease |
| L40 | Psoriasis | Autoimmune disease |
| L63 | Alopecia areata | Autoimmune disease |
| L80 | Vitiligo | Autoimmune disease |
| M02 | Reactive arthropathies | Autoimmune disease |
| M05 | Seropositive rheumatoid arthritis | Autoimmune disease |
| M06 | Other rheumatoid arthritis | Autoimmune disease |
| M08 | Juvenile arthritis | Autoimmune disease |
| M32 | Systemic lupus erythematosus | Autoimmune disease |
| M33 | Dermatopolymyositis | Autoimmune disease |
| M34 | Systemic sclerosis | Autoimmune disease |
| M45 | Ankylosing spondylitis | Autoimmune disease |
| I20 | Angina pectoris | Cardio- and cerebrovascular disease |
| I21 | Acute myocardial infarction | Cardio- and cerebrovascular disease |
| I22 | Subsequent myocardial infarction | Cardio- and cerebrovascular disease |
| I23 | Certain current complications following acute myocardial infarction | Cardio- and cerebrovascular disease |
| I24 | Other acute ischaemic heart diseases | Cardio- and cerebrovascular disease |
| I25 | Chronic ischaemic heart disease | Cardio- and cerebrovascular disease |
| I63 | Cerebral infarction | Cardio- and cerebrovascular disease |
| I64 | Stroke, not specified as haemorrhage or infarction | Cardio- and cerebrovascular disease |
| I65 | Occlusion and stenosis of precerebral arteries, not resulting in cerebral infarction | Cardio- and cerebrovascular disease |
| I66 | Occlusion and stenosis of cerebral arteries, not resulting in cerebral infarction | Cardio- and cerebrovascular disease |
| R45 | Symptoms and signs involving emotional state | Self-harming behaviour not resulting in death |
| X71 | Intentional self-harm by drowning and submersion | Self-harming behaviour not resulting in death |
| X72 | Intentional self-harm by handgun discharge | Self-harming behaviour not resulting in death |
| X73 | Intentional self-harm by rifle, shotgun and larger firearm discharge | Self-harming behaviour not resulting in death |
| X74 | Intentional self-harm by other and unspecified firearm discharge | Self-harming behaviour not resulting in death |
| X75 | Intentional self-harm by explosive material | Self-harming behaviour not resulting in death |
| X76 | Intentional self-harm by smoke, fire and flames | Self-harming behaviour not resulting in death |
| X77 | Intentional self-harm by steam, hot vapours and hot objects | Self-harming behaviour not resulting in death |
| X78 | Intentional self-harm by sharp object | Self-harming behaviour not resulting in death |
| X80 | Intentional self-harm by jumping from a high place | Self-harming behaviour not resulting in death |
| X81 | Intentional self-harm by jumping or lying before moving object | Self-harming behaviour not resulting in death |
| X82 | Intentional self-harm by crashing of motor vehicle | Self-harming behaviour not resulting in death |
| X83 | Intentional self-harm by other specified means | Self-harming behaviour not resulting in death |
| T36 | Poisoning by systemic antibiotics | Self-harming behaviour not resulting in death |
| T37 | Poisoning by other systemic anti-infectives and antiparasitics | Self-harming behaviour not resulting in death |
| T38 | Poisoning by hormones and their synthetic substitutes and antagonists, not elsewhere classified | Self-harming behaviour not resulting in death |
| T39 | Poisoning by non-opioid analgesics, antipyretics and antirheumatics | Self-harming behaviour not resulting in death |
| T40 | Poisoning by narcotics and psychodysleptics (hallucinogens) | Self-harming behaviour not resulting in death |
| T41 | Poisoning by anaesthetics and therapeutic gases | Self-harming behaviour not resulting in death |
| T42 | Poisoning by antiepileptic, sedative-hypnotic and antiparkinsonism drug) | Self-harming behaviour not resulting in death |
| T43 | Poisoning by psychotropic drugs, not elsewhere classified | Self-harming behaviour not resulting in death |
| T44 | Poisoning by drugs primarily affecting the autonomic nervous system | Self-harming behaviour not resulting in death |
| T45 | Poisoning by primarily systemic and haematological agents, not elsewhere classified | Self-harming behaviour not resulting in death |
| T46 | Poisoning by agents primarily affecting the cardiovascular system | Self-harming behaviour not resulting in death |
| T47 | Poisoning by agents primarily affecting the gastrointestinal system | Self-harming behaviour not resulting in death |
| T48 | Poisoning by agents primarily acting on smooth and skeletal muscles and the respiratory system | Self-harming behaviour not resulting in death |
| T49 | Poisoning by topical agents primarily affecting skin and mucous membrane and by ophthalmological, otorhinolaryngological and dental drugs | Self-harming behaviour not resulting in death |
| T50 | Poisoning by diuretics and other unspecified drugs, medicaments and biological substances | Self-harming behaviour not resulting in death |
| Y22 | Handgun discharge, undetermined intent | Self-harming behaviour not resulting in death |
| Y23 | Rifle, shotgun and larger firearm discharge, undetermined intent | Self-harming behaviour not resulting in death |
| Y24 | Other and unspecified firearm discharge, undetermined intent | Self-harming behaviour not resulting in death |
| Y25 | Contact with explosive material, undetermined intent | Self-harming behaviour not resulting in death |
| Y26 | Exposure to smoke, fire and flames, undetermined intent | Self-harming behaviour not resulting in death |
| Y27 | Contact with steam, hot vapours and hot objects, undetermined intent | Self-harming behaviour not resulting in death |
| Y28 | Contact with sharp object, undetermined intent | Self-harming behaviour not resulting in death |
| Y30 | Falling, jumping or pushed from a high place, undetermined intent | Self-harming behaviour not resulting in death |
| Y31 | Falling, lying or running before or into moving object, undetermined intent | Self-harming behaviour not resulting in death |
| Y32 | Crashing of motor vehicle, undetermined intent | Self-harming behaviour not resulting in death |
| Y33 | Other specified events, undetermined intent | Self-harming behaviour not resulting in death |

ICD, International Classification of Diseases and Related Health Problems.
