# Supplementary material for: Clinical characteristics of treatment-resistant depression in adults in Hungary: Real-world evidence from a 7-year-long retrospective data analysis
Source: PLoS One. 2021 Jan 20;16(1):e0245510. doi: 10.1371/journal.pone.0245510 (PMC7817060; doi:10.1371/journal.pone.0245510)
Supplement: S3 File — (DOCX) [file pone.0245510.s003.docx]

**S7 Appendix. Categorisation of AD and add-on drugs used in the study based on the Hungarian reimbursement guidelines.**

The Decree Nr. 32/2004. (IV. 26.) of the Ministry of Health and Welfare [1] details the way different drugs are categorised for reimbursement in Hungary.

One of the sections cover the reimbursement of medications used in the treatment of “Mood, neurotic, stress-related, somatoform disorders, and bulimia nervosa” which includes but is not limited to antidepressants. Not all drugs listed in this section are identified as an AD or add-on medication in our study. These drugs are excluded from this appendix.

The section outlines three categories of reimbursement: EÜ90 7/a1, EÜ90 7/a2, EÜ90 7/a3. It mandates that any drugs reimbursed under EÜ90 7/a2 can only be reimbursed for patients who had documented failure of treatment with drugs reimbursed under EÜ90 7/a1. It also mandates that any drugs reimbursed under EÜ90 7/a3 can only be reimbursed for patients who had documented failure of treatment with drugs reimbursed under EÜ90 7/a2.

For the sake of brevity drugs reimbursed under EÜ90 7/a1, EÜ90 7/a2, EÜ90 7/a3 were denoted as Category 1, Category 2 and Category 3, respectively.

The continuously updated list of medications belonging to each category can be found on the homepage of the NHIF (neak.gov.hu).

The following table contains the drugs belonging to the three categories which were classified as either ADs or add-on medications in the study.

**S8 Table. List of drugs belonging to each category.**

| **ATC code** | **Active substance** | **Category** |
| --- | --- | --- |
| N03AE01 | clonazepam | Category 1 |
| N06AA02 | imipramine | Category 1 |
| N06AA04 | clomipramine | Category 1 |
| N06AA09 | amitriptyline | Category 1 |
| N06AA21 | maprotiline | Category 1 |
| N06AB03 | fluoxetine | Category 1 |
| N06AB04 | citalopram | Category 1 |
| N06AB05 | paroxetine | Category 1 |
| N06AB06 | sertraline | Category 1 |
| N06AB08 | fluvoxamine | Category 1 |
| N06AG02 | moclobemide | Category 1 |
| N05AL03 | tiapride | Category 2 |
| N06AB10 | escitalopram | Category 2 |
| N06AX03 | mianserin | Category 2 |
| N06AX05 | trazodone | Category 2 |
| N06AX11 | mirtazapine | Category 2 |
| N06AX14 | tianeptine | Category 2 |
| N06AX18 | reboxetine | Category 3 |
| N06AX21 | duloxetine | Category 3 |
| N06AX22 | agomelatine | Category 3 |
| N06AX26 | vortioxetine | Category 3 |

ATC, Anatomical Therapeutic Chemical.

[1] 32/2004. (IV. 26.) ESzCsM rendelet a törzskönyvezett gyógyszerek és a különleges táplálkozási igényt kielégítő tápszerek társadalombiztosítási támogatásba való befogadásának szempontjairól és a befogadás vagy a támogatás megváltoztatásáról <http://njt.hu/cgi_bin/njt_doc.cgi?docid=84085.382508> Hungarian.
